# Evaluation of Choroidal Structure in Type 1 Macular Neovascularization Using Different Optical Coherence Tomography Analyses: Scale Bar and Binarization

**DOI:** 10.3390/jcm13051383

**Published:** 2024-02-28

**Authors:** Hiromasa Hirai, Mariko Yamashita, Nobuo Ijuin, Hironobu Jimura, Tomo Nishi, Nahoko Ogata, Tetsuo Ueda

**Affiliations:** 1 Department of Ophthalmology, Nara Medical University, 840 Shijo-cho, Kashihara 634-8521, Japan; 2 Department of Ophthalmology, Nara Prefecture Seiwa Medical Center, 1-14-16 Mimuro, Sango 636-0802, Japan; 3 Department of Ophthalmology, Nara City Hospital, 1-50-1 Higashikidera-cho, Nara 630-8305, Japan

**Keywords:** age-related macular degeneration, choroidal neovascularization, macular neovascularization, binarization, the ratio of the luminal to the choroidal area, choroidal vascularity index

## Abstract

Background: Macular neovascularization (MNV) has been evaluated by optical coherence tomography (OCT) imaging using various approaches. However, few studies have examined their differences. This study analyzed type 1 MNV with a combination of two approaches: scale bar and binarization. Methods: We enrolled 84 patients with untreated type 1 MNV. We measured choroidal parameters using a scale bar and defined the ratios of superficial choroidal thickness to choroidal vessel diameter (SV ratios). We also used binarization and calculated the ratios of the luminal to the choroidal area (LC ratios) in two directions (horizontal and vertical). Results: Fifty-one patients (61%) were classified as having polyps. SV ratios in the group with polyps were significantly lower than in the group without (*p* < 0.001). The receiver operating characteristic (ROC) curve showed that the SV ratio was predictive of polyps (AUC 0.733, 95% CI: 0.621–0.844). In patients without polyps, horizontal LC ratios were significantly higher in a subgroup with subretinal fluid than in those without (*p* = 0.047). The ROC curve showed that the LC ratio was predictive of subretinal fluid (AUC 0.722, 95% CI: 0.517–0.926). Conclusion: The SV ratio reflects the MNV disease type, whereas the LC ratio reflects MNV disease activity. Establishing cut-off values for each ratio may be useful for MNV diagnosis.

## 1. Introduction

Macular neovascularization (MNV) in age-related macular degeneration typically develops beneath the fovea and can cause serious visual impairment in the elderly [1,2]. MNVs are classified into type 1 (under the retinal pigment epithelium [RPE]) or type 2 (above the RPE) based on their location in optical coherence tomography (OCT) images [3]. In type 1 MNV, polypoidal lesions are often observed on the indocyanine green angiography (ICGA) [3,4]. Subretinal fluid (SRF) is frequently recognized as a hyporeflective space between the retina and the RPE in OCT images [3]. SRF is considered an important indicator for evaluating the disease activity of MNV [5,6].

To assess the pathological lesions of MNV, the choroidal structure was evaluated in OCT images using various methods. Several researchers have analyzed the choroidal thickness in each layer using a scale bar in the OCT systems [7,8,9]. Recently, ratios of the luminal to the choroidal area (LC ratios, also known as choroidal vascularity index) based on binarized images have been proposed, and several studies have used this binarization method [10,11,12,13,14]. However, few studies have compared these OCT evaluations. Furthermore, their cut-off values were unclear. Therefore, this study aimed to investigate the choroidal structure of type 1 MNV by combining two approaches: scale bar and binarization.

## 2. Materials and Methods

This retrospective cross-sectional study included patients who visited the Department of Ophthalmology, Nara Medical University, Kashihara City, Nara Prefecture, Japan, between 2012 and 2018. Ophthalmological examinations such as slit-lamp examination, fundus examination, SD-OCT (Spectralis, Heidelberg Engineering, Heidelberg, Germany), fundus photography, fluorescein angiography (FA), and ICGA were performed in all patients at the initial visit. We obtained two OCT images by using a single line of 9 mm length and an average of 49 B-scans from two directions (horizontal and vertical). We also used enhanced depth imaging (EDI)-OCT to obtain fine images of the choroidal structures. All patients were treatment-naïve and were diagnosed with type 1 MNV.

**Figure 1:**
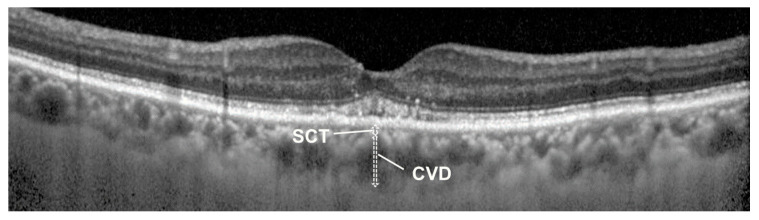
Measuring of superficial choroidal thickness (SCT) and choroidal vessel diameter (CVD) in an OCT image of 75-year-old male. CVD is defined as the thickness of the largest hyporeflective lumen within Haller’s layer. SCT is defined as the distance from the bottom of the RPE to above the choroidal vessel where the CVD measurement was taken, encompassing the combined thickness of the choriocapillaris and Sattler’s layer. Each choroidal parameter was measured from horizontal and vertical OCT images, and the mean value for each patient was calculated. The SV ratio, which represents the ratio of mean SCT to mean CVD, was defined as a relative value.

We defined type 1 MNV as early leakage in the FA and hyperreflective lesions under the RPE using OCT images based on previous studies [3,15]. Polyp lesions were detected using ICGA. We excluded patients with inadequate choroidal thickness measurements, history of intraocular inflammation within the past three months, severe myopia, progressive diabetic retinopathy, and severe glaucoma. After exclusion, we enrolled 84 patients with type 1 MNV. The presence of SRF was defined in OCT as a hyporeflective space between the retina and the RPE. Patients with hyporeflective space on either of the two scan images were assigned to the group with SRF. The pigment epithelial detachment (PED) and drusen were also evaluated using fundus photography and OCT images. We measured central choroidal thickness (CCT), choroidal vessel diameter (CVD), and superficial choroidal thickness (SCT) using EDI-OCT images. Based on previous studies [7,8,16,17], each parameter was defined as follows (Figure 1): choroidal thickness beneath the fovea (CCT), thickness of the largest hyporeflective lumen in the Haller’s layer (CVD), and distance from the bottom of the RPE to above the hyporeflective lumen where CVD was measured (SCT). SCT was regarded as the combined thickness of the choriocapillaris and Sattler’s layer.

**Figure 2:**
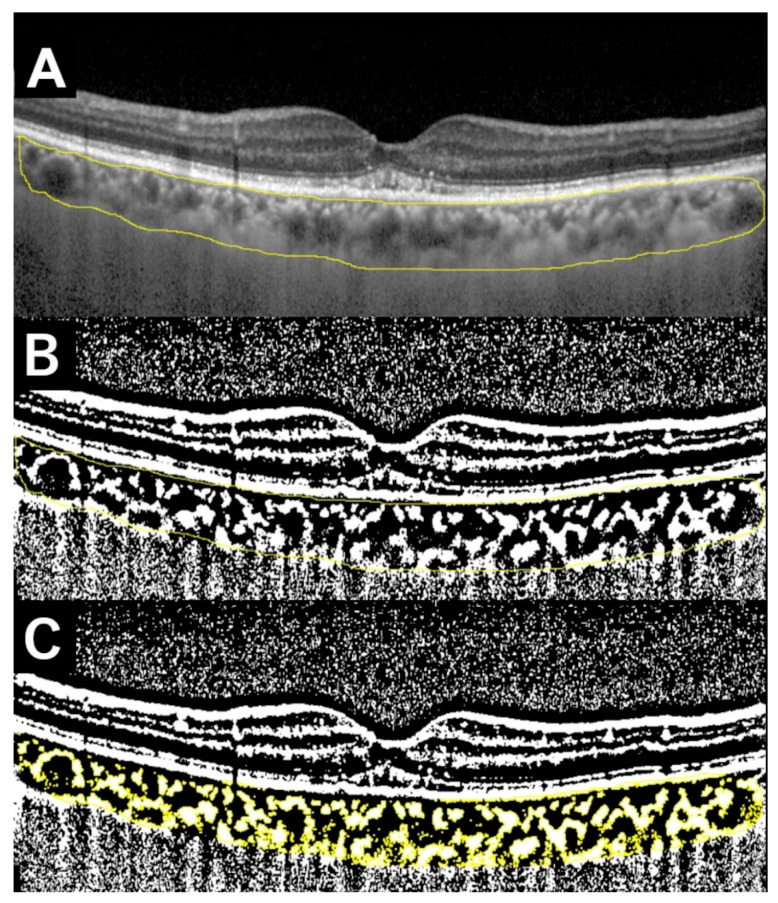
The process of measuring the ratio of the luminal to the choroidal area (LC ratio) in an OCT image of a 75-year-old male (same as Figure 1). (**A**) Specify the entire choroid area within the arcade (inside the yellow line). (**B**) Binarize the image to black and white using Niblack method. The white area is considered as the interstitial component whereas the black area as the luminal component. (**C**) Specify the interstitial area (colored yellow) and calculate the interstitial area and total area, respectively. The LC ratio is calculated as 100 − (interstitial area/total area) × 100 (%). In this example, the LC ratio is 63.9%.

The CCT and two values (CVD and SCT) were measured independently by different researchers using a scale bar included in the OCT system. For accuracy, we measured each choroidal parameter from horizontal and vertical OCT images and calculated the mean value for each patient. We validated the manual choroidal parameter measurements by using Bland–Altman analysis to check the agreement of two measurements [18]. We further calculated intraclass correlation coefficients (ICC) to examine test reliability based on the previous study [19]. The SV ratio, indicating the ratio of mean SCT to mean CVD, was defined as a relative value. We also binarized the OCT images by Niblack method using image analysis software (Fiji ImageJ, version 1.53t, Java 1.8.0_322 [64-bit], National Institutes of Health, Bethesda, Maryland, USA) and calculated the LC ratios based on previous studies [10,20,21,22,23] (Figure 2).

We designated the LC ratio calculated from the horizontal OCT scan as the horizontal LC ratio, and the ratio obtained from the vertical OCT scan as the vertical LC ratio. Correlations were evaluated using Pearson’s correlation coefficients. We used *t*-tests and Mann–Whitney U tests to compare the averages of continuous variables, while Fisher’s exact tests were employed for categorical variables between the two groups. Receiver operating characteristic (ROC) curve analysis, along with the calculation of the area under the curve (AUC), was performed to classify the groups. Cut-off values were defined using the Youden index [24]. Statistical analyses were conducted using EZR (Saitama Medical Center, Jichi Medical University, Saitama, Japan), a graphical user interface for R (R Foundation for Statistical Computing, Vienna, Austria) [25]. The threshold for statistical significance was set at *p* < 0.05.

An opt-out method was used to obtain informed consent. The opt-out form adequately described the intention and purpose of this study. The study was approved by the Institutional Review Board of Nara Medical University (protocol code: 2107) and was conducted in accordance with the Declaration of Helsinki. All methods were performed according to the relevant guidelines and regulations.

## 3. Results

The characteristics of patients are presented in Table 1.

The median age of the 84 patients was 72.5 years. Among them, 61 (73%) were male. The median CCT was 228 μm. Fifty-one patients (61%) were categorized as having polyps, while 63 patients (75%) had SRF. Table 2 shows the characteristics of patients separated by gender. There was no significant difference between the two groups.

**Figure 3:**
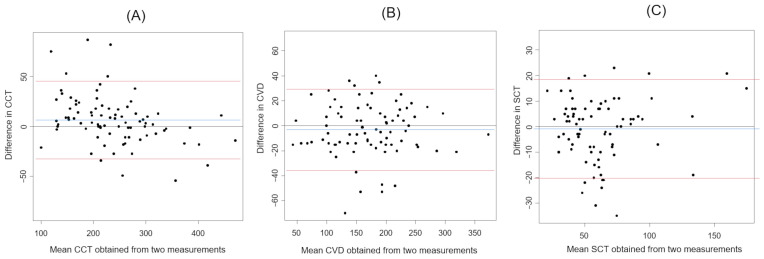
The Bland–Altman plots of each choroidal parameter measured by the scale bar. (**A**) CCT. (**B**) CVD. (**C**) SCT. Blue lines represent the mean difference. Red lines represent 95% limits of agreement.

The results of the Bland–Altman analysis with three values (CCT, CVD, and SCT) measured by a scale bar are shown in Figure 3.

The results of ICC are summarized in Table 3. Each ICC showed high reliability.

**Figure 4:**
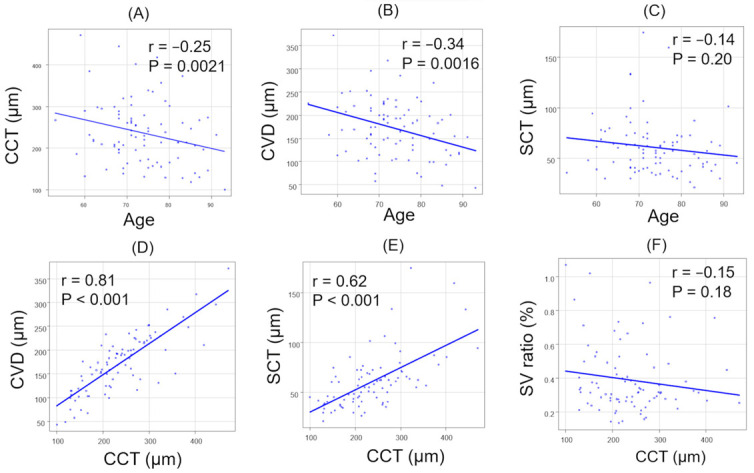
The correlation between age and each parameter (**A**–**C**), and between CCT and each parameter (**D**–**F**). (**A**) Age and CCT. (**B**) Age and CVD. (**C**) Age and SCT. (**D**) CCT and CVD. (**E**) CCT and SCT. (**F**) CCT and SV ratio.

The correlations between age and each parameter in all patients are summarized (Figure 4A–C). There were significant negative correlations in CCT (r = −0.25, *p* = 0.0021) and CVD (r = −0.34, *p* = 0.0016). In contrast, no significant correlation was observed between age and SCT (r = −0.14, *p* = 0.20). The correlations between CCT and other parameters in all patients are summarized (Figure 4D–F). There was a significant positive correlation between CCT and CVD (r = 0.81, *p* < 0.001).

**Figure 5:**
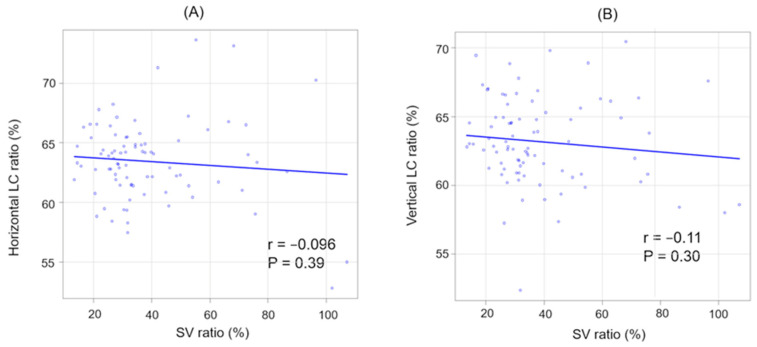
The correlation between SV and LC ratios (horizontal and vertical). (**A**) SV ratio and horizontal LC ratio. (**B**) SV ratio and vertical LC ratio.

In addition, there was a significant positive correlation between CCT and SCT (r = 0.62, *p* < 0.001). However, no significant correlation was observed between CCT and the SV ratio (r = −0.15, *p* = 0.18). Across all patients, there were no significant correlations between the SV and LC ratios in either direction (Figure 5).

**Figure 6:**
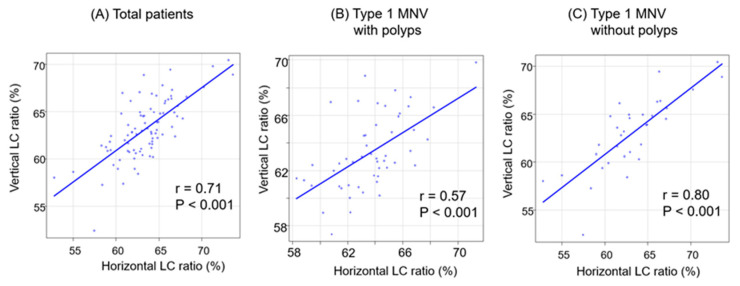
The correlation between horizontal and vertical LC ratios classified by polyps (**A**–**C**). (**A**) Total patients. (**B**) Type 1 MNV with polyps. (**C**) Type 1 MNV without polyps.

The correlations between the LC ratios in the two directions in each group are illustrated in Figure 6.

In all the groups, significant positive correlations were observed in the LC ratios between the two directions. The positive correlation was strongest in the group without polyps (r = 0.80, *p* < 0.001).

Comparisons of the patients classified according to the presence of polyps are presented in Table 4.

**Figure 7:**
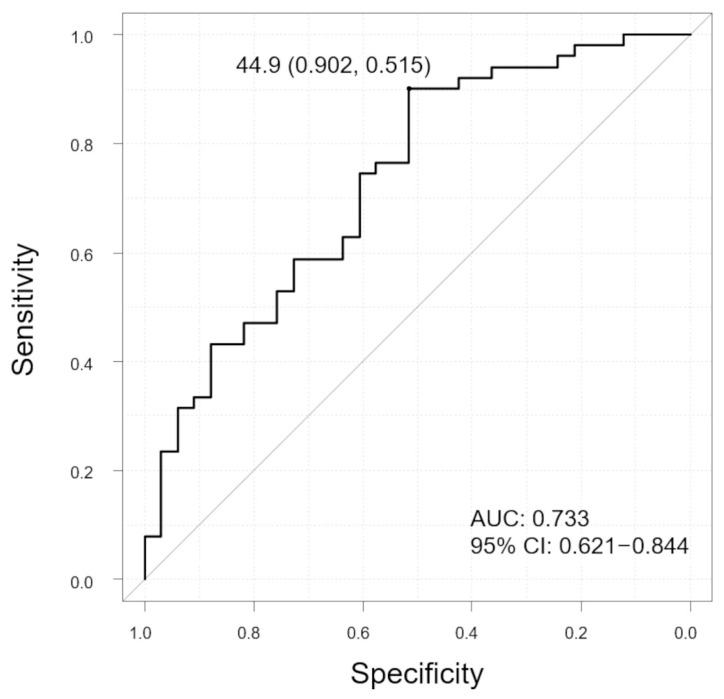
Receiver operating characteristic (ROC) curve to evaluate the ability of SV ratio to distinguish between polyp and non-polyp groups in all patients. The graph displays the cutoff value (sensitivity, specificity). Vertical axis of the graph, sensitivity; horizontal axis of the graph, specificity; AUC, area under the curve; and CI, confidence interval.

SCT was significantly thinner in cases with polyps than in those without polyps (*p* = 0.0047). The SV ratios were also significantly lower in cases with polyps than in those cases without polyps (*p* < 0.001). In contrast, there was no significant difference in CVD incidence between the two groups (*p* = 0.12). In addition, no significant difference was observed in the horizontal and vertical LC ratios (*p* = 0.48, 0.67, respectively). The ROC curve showed that the SV ratio was predictive of polyp (Figure 7, AUC 0.733, 95% CI: 0.621–0.844). The sensitivity and specificity were 90.2% and 51.5%, respectively, with the SV ratio cutoff value of 44.9%.

We also compared patients classified according to the presence of SRF in each subgroup (Table 5).

**Figure 8:**
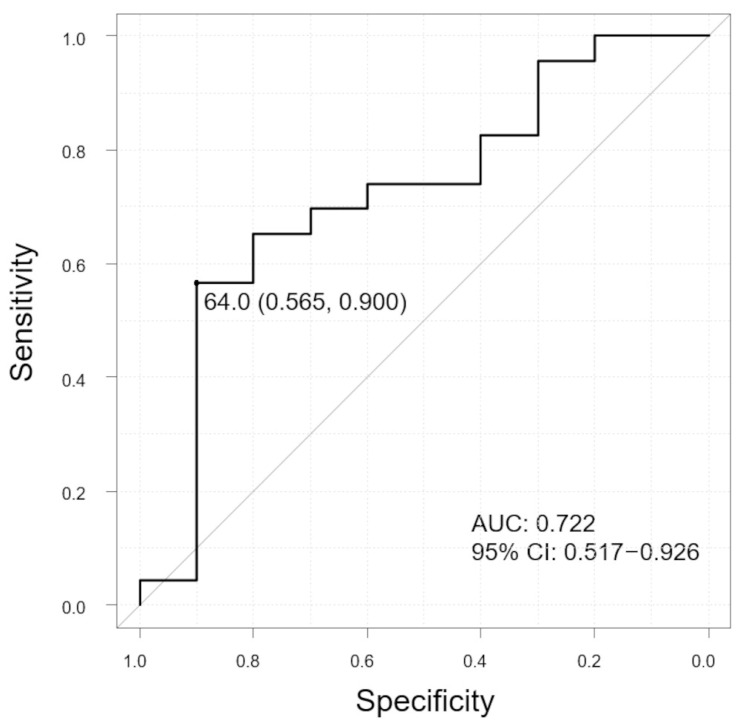
ROC curve to evaluate the ability of LC ratio to distinguish between SRF and non-SRF groups in patients without polyps. The graph shows the cutoff value (sensitivity, specificity). Vertical axis of the graph, sensitivity; horizontal axis of the graph, specificity; and AUC, area under the curve; CI, confidence interval.

In the group without polyps, the horizontal LC ratios were significantly higher in the subgroup with SRF than in the subgroup without SRF (*p* = 0.047). However, no significant differences were observed in the group with polyps. The ROC curve showed that the LC ratio was predictive of SRF (Figure 8, AUC 0.722, 95% CI: 0.517–0.926). The sensitivity and specificity were 56.5% and 90.0%, respectively, with the LC-ratio cut-off value of 64.0%.

## 4. Discussion

This study investigated the choroidal structure of type 1 MNV through a combined utilization of scale bar and binarization methodologies. In the present study, the patient group included a larger number of males. This result may be attributed to the large number of cases with polyps. It has been known that polyp formation in MNV is more common in Asian males [4]. We examined the characteristics of patients separated by gender and confirmed no significant differences. A margin of error can become a problem when choroidal parameters are measured manually. Therefore, we used Bland–Altman analysis to validate the errors between two measurements based on the previous studies [26,27]. The results showed that the errors of CVD and SCT tended to be smaller than those of CCT. The ICC results also indicate the reliability of the present measurements. In the correlation analysis of each parameter, age exhibited a significant negative correlation with CCT and CVD across all patients. However, no significant relationship was observed between age and SCT. Several studies have reported thinning of the Haller’s layer with age [8,28]. In patients with type 1 MNV, the reduction in CVD may be more pronounced in older patients, potentially impacting CCT. Previous studies evaluated choroidal parameters as absolute values, preventing comparisons between patients [16,29]. Therefore, in this study, we defined the SV ratio (ratio of SCT to CVD) as a relative value. While both CVD and SCT significantly correlated with CCT, there was no correlation between the SV ratio and CCT. This suggests the independence of the SV ratio.

Additionally, SV ratios (based on a scale bar) were observed to be lower in the group with polyps compared to the group without polyps. Our results also showed that the SCT was significantly thinner in patients with polyps. It has been reported that the choriocapillaris and Sattler layers are attenuated at the site of polyp lesions, while the Haller layer vessels are dilated [30].

On the other hand, there was no significant difference in CVD between the two groups in our study. A recent study suggested that decreased vascular density in the superficial choroid is an independent risk factor for polyp development [31]. Thinning of the superficial layer may be responsible for the reduced vascular density. Our results revealed a lower SV ratio in patients with polyps, suggesting greater superficial compaction.

We also calculated the LC ratio based on the binarized image. LC ratio and choroidal thickness are known to increase with exudative macular changes [32]. While choroidal thickness is known to be affected by age, LC ratios have been reported to be constant in healthy subjects regardless of age [33]. A recent study has proposed that polyp lesions act as an arteriovenous shunt in the choroid, resulting in an increased interstitial component and a smaller LC ratio [34]. However, LC ratios did not differ significantly between two groups classified by polyps in the present study. In the LC ratio, the low-signal portion is recognized as the vascular component [10,35]. Together with the results of a low SV ratio (measured by scale bar) in cases with polyps, the proportion of focal choroidal vessels may differ from an entire region in cases with polyps. Our results suggest that focal choroidal vessels may be found in shallow locations in patients with polyps.

In contrast, the horizontal LC ratios in patients without polyps were significantly higher in the subgroup with SRF than in the subgroup without SRF. SRF is an outflow from the choroid due to RPE dysfunction [36]. A larger choroidal luminal area in patients without polyps may make them more prone to exudative changes. In contrast, the difference in the LC ratios was not significant between the two subgroups of polyps classified by the presence of SRF. This result might be derived from the fact that the presence of polyps affects the exudative formation.

We also examined LC ratios using scan direction. Few studies have examined the differences in LC ratios by scanning the direction of OCT. Although a recent study reported differences in LC ratios between scanning directions in young healthy subjects [37], our results suggest that the choroidal lumen distribution may remain uniform between the horizontal and vertical directions. Although the vertical LC ratio showed no significant difference in the presence of SRF, this result might be due to the relatively small sample size.

Moreover, we also calculated AUC and were able to set the cut-off values. Few studies have mentioned cut-off values for those ratios. These cut-off values showed different characteristics: the SV ratio had high sensitivity, while the LC ratio had high specificity. Our results suggest that the SV ratio is useful for screening eyes that may develop MNVs with polyps, whereas the LC ratio is useful for identifying MNVs that may form SRF. Together with the result that there was no apparent correlation between the SV and LC ratios, these two ratios reflect different choroidal features. A recent study reported that polyp lesions have transformed into type 1 MNV after anti-VEGF treatment [38]. This result suggests the change of disease type during the long period. Thus, a combination of both approaches (scale bar and binarization) may be useful for evaluating type 1 MNV.

This study has several limitations. First, we performed the measurements on a relatively small number of samples. Therefore, it is desirable to study larger sample sizes. Second, we measured only the first pretreatment patients; therefore, the long-term outcomes are unknown. Further studies with long-term observations are needed.

## 5. Conclusions

In summary, we obtained unique results focusing on choroidal parameters in patients with type 1 MNV. Our results suggest an association between the SV ratio and MNV disease type, whereas the LC ratio may be helpful for evaluating MNV disease activity. The establishment of cut-off values for each ratio may be useful for MNV diagnosis.

## Figures and Tables

**Table 1:** The characteristics of patients (n = 84).

Age, median (IQR)	72.5 (68.0–79.0)
Sex (male), n (%)	61 (73)
Polyp lesions, n (%)	51 (61)
BCVA (LogMAR unit), Median (IQR)	0.19 (0.05–0.40)
SRF, n (%)	63 (75)
PED, n (%)	28 (33)
Drusen, n (%)	18 (21)
CCT, median (IQR), µm	228 (183–275)
CVD, median (IQR), µm	172 (125–212)
SCT, median (IOR), µm	57.0 (40.9–71.1)
SV ratio (%), median (IQR)	31.7 (26.7–45.9)
Horizontal LC ratio (%), Median (IQR)	63.3 (61.5–65.2)
Vertical LC ratio (%), Median (IQR)	62.8 (61.0–65.0)

Abbreviations: IQR, inter-quartile range; BCVA, best-corrected visual acuity (LogMAR unit); SRF, subretinal fluid; PED, pigment epithelium detachment; CCT, central choroidal thickness; CVD, choroidal vessel diameter; SCT, superficial choroidal thickness; SV ratio, the ratio of SCT to CVD; and LC ratio, the ratio of the luminal to the choroidal area.

**Table 2:** The characteristics of patients separated by gender.

	Male (n = 61)	Female (n = 23)	*p* Value
Age, median (IQR)	74.0 (69.0–79.0)	69.0 (64.5–78.5)	0.19
Polyp lesions, n (%)	38 (62)	13 (57)	0.63
SRF, n (%)	48 (79)	15 (65)	0.26
PED, n (%)	17 (28)	11 (48)	0.12
Drusen, n (%)	16 (26)	2 (9)	0.13

Abbreviations: IQR, inter-quartile range; SRF, subretinal fluid; and PED, pigment epithelium detachment. All *p* values were calculated by the Fisher’s exact test.

**Table 3:** The Intraclass correlation coefficients (ICC) of each choroidal parameter.

	CCT	CVD	SCT
ICC (1,1), 95%CI	0.947 (0.920–0.965)	0.945 (0.917–0.964)	0.907 (0.861–0.939)
ICC (1,2), 95%CI	0.973 (0.958–0.982)	0.972 (0.957–0.982)	0.951 (0.925–0.969)

Abbreviations: CCT, central choroidal thickness; CVD, choroidal vessel diameter; SCT, superficial choroidal thickness; and CI, confidence interval.

**Table 4:** Comparisons of patients classified by the presence of polyps.

	Type1 MNV without Polyps (n = 33)	Type1 MNV with Polyps (n = 51)	*p* Value
Age, median (IQR)	74.0 (68.0–80.0)	72.0 (68.0–78.0)	0.27 †
Sex (male), n (%)	23 (70)	38 (75)	0.63 ‡
BCVA (LogMAR unit), Median (IQR)	0.15 (0.10–0.40)	0.22 (0.05–0.40)	0.74 †
SRF, n (%)	23 (70)	40 (78)	0.44 ‡
PED, n (%)	7 (21)	21 (41)	0.06 ‡
Drusen, n (%)	6 (18)	12 (24)	0.6 ‡
CCT, Median (IQR), µm	248 (206–289)	214 (174–261)	0.56 †
CVD, Median (IQR), µm	158 (116–215)	175 (147–209)	0.12 †
SCT, Median (IOR), µm	63.5 (57.0–72.5)	48.5 (37.5–63.8)	0.0047 †
SV ratio (%), Median (IQR)	45.8 (31.1–63.0)	30.4 (24.5–36.4)	<0.001 †
Horizontal LC ratio (%), Median (IQR)	62.8 (60.6–65.5)	63.9 (62.1–65.0)	0.48 †
Vertical LC ratio (%), Median (IQR)	63.6 (60.6–65.0)	62.7 (61.3–65.1)	0.67 †

Abbreviations: IQR, inter-quartile range; MNV, macular neovascularization; BCVA, best-corrected visual acuity (LogMAR unit); SRF, subretinal fluid; PED, pigment epithelium detachment; CCT, central choroidal thickness; CVD, choroidal vessel diameter; SCT, superficial choroidal thickness; SV ratio, the ratio of SCT to CVD; and LC ratio, the ratio of the luminal to the choroidal area. † *t* test; ‡ Fisher’s exact test.

**Table 5:** Comparison of patients classified by the presence of SRF in each group.

	Type1 MNV without Polyps (n = 33)		*p* Value	Type1 MNV with Polyps (n = 51)		*p* Value
	SRF (+) (n = 23)	SRF (−) (n = 10)		SRF (+) (n = 40)	SRF (−) (n = 11)	
CCT, median (IQR), µm	256 (214–294)	229 (165–278)	0.34	220 (183–263)	209 (132–237)	0.27
CVD, median (IQR), µm	176 (132–221)	119 (82–183)	0.06	183 (153–212)	150 (110–203)	0.2
SCT, median (IOR), µm	65.5 (56.0–72.0)	63.0 (57.8–74.3)	0.85	49.3 (37.9–68.8)	46.5 (31.8–58.5)	0.28
SV ratio (%), median (IQR)	36.5 (29.9–53.9)	58.5 (46.8–81.9)	0.07	28.9 (24.8–36.1)	31.8 (25.1–37.1)	0.69
Horizontal LC ratio (%), Median (IQR)	64.2 (61.7–66.2)	61.6 (58.9–62.5)	0.047	63.5 (62.1–64.8)	64.2 (61.2–65.1)	0.9
Vertical LC ratio (%), Median (IQR)	64.5 (61.7–65.3)	60.5 (58.5–63.5)	0.08	62.8 (61.5–65.0)	62.2 (61.3–64.5)	0.63

Abbreviations: SRF, subretinal fluid; IQR, inter-quartile range; MNV, macular neovascularization; CCT, central choroidal thickness; CVD, choroidal vessel diameter; SCT, superficial choroidal thickness; SV ratio, the ratio of SCT to CVD; and LC ratio, the ratio of the luminal to the choroidal area. All *p* values were calculated by the Mann–Whitney U test.

## Data Availability

The data presented in this study are available on request from the corresponding author on reasonable request.
